# A Plasmacytoid Variant of Urothelial Carcinoma: A Rare Entity

**DOI:** 10.7759/cureus.67436

**Published:** 2024-08-21

**Authors:** Ammara Yasmin, Midhat Waheed, Muhammad Ahsan Jamil, Maryam Imran, Muhammad Awais Majeed

**Affiliations:** 1 Oncology, Shaukat Khanum Memorial Cancer Hospital and Research Centre, Lahore, PAK; 2 Medical Oncology, Shaukat Khanum Memorial Cancer Hospital and Research Centre, Lahore, PAK

**Keywords:** chemotherapy, infiltrative, ecadherin, plasmacytoid, urothelial carcinoma

## Abstract

A rare histological variant of transitional cell urothelial carcinoma, the plasmacytoid variant, was recently included in the World Health Organization classification of urothelial tract tumors. This variant has a morphological resemblance to other tumors, which poses a diagnostic challenge for identifying this tumor and may often lead to misdiagnosis. Vigilant histopathological analysis and immunostaining are required to delineate the correct diagnosis. The plasmacytoid variant of urothelial carcinoma is an aggressive tumor with a poor prognosis, making correct diagnosis essential for early and appropriate treatment. This paper presents the case of a 46-year-old male with a plasmacytoid variant of high-grade urothelial carcinoma who underwent transurethral resection of a bladder tumor, received chemotherapy, and is currently undergoing follow-up.

## Introduction

Bladder cancer is one of the most common cancers worldwide. Men are four times more prone to developing bladder carcinoma than females [1-4]. Moreover, urothelial carcinomas more frequently occur in elderly people with multiple comorbidities, creating treatment challenges. Urothelial carcinomas comprise 90% of bladder tumors and have divergent differentiation.

In 1991, Sahin et al. described a new variant of bladder cancer simulating lymphoma and plasmacytoma, plasmacytoid urothelial carcinoma (PUC), which is a rare entity with unique clinical features and aggressive behavior and is also known as poorly cohesive or diffuse carcinoma [5]. The World Health Organization (WHO) classification 2004 recognizes several morphological variants of urothelial carcinomas, and plasmacytoid variants are defined as one of the subcategories of infiltrating urothelial tumors [6]. The use of immunohistochemistry for pathological diagnosis is important for obtaining a correct diagnosis, and the consideration of this variant by pathologists and urologists is important for avoiding misdiagnosis. Here, we report a rare plasmacytoid variant of urothelial carcinoma in a 46-year-old male who was treated and remains under follow-up.

## Case presentation

**Figure 1:**
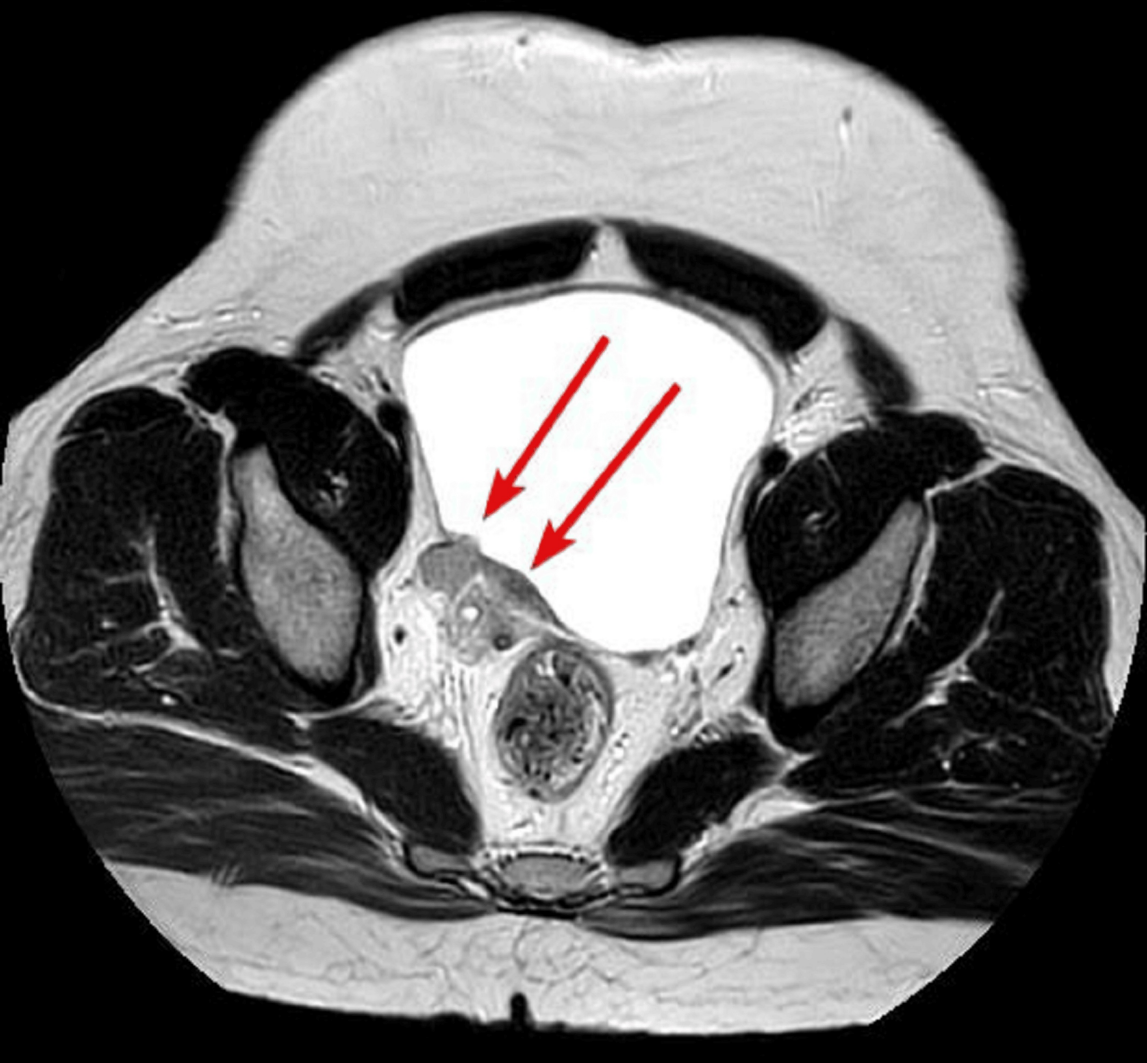
Bladder wall lesion visualized on MRI T2 images.

**Figure 2:**
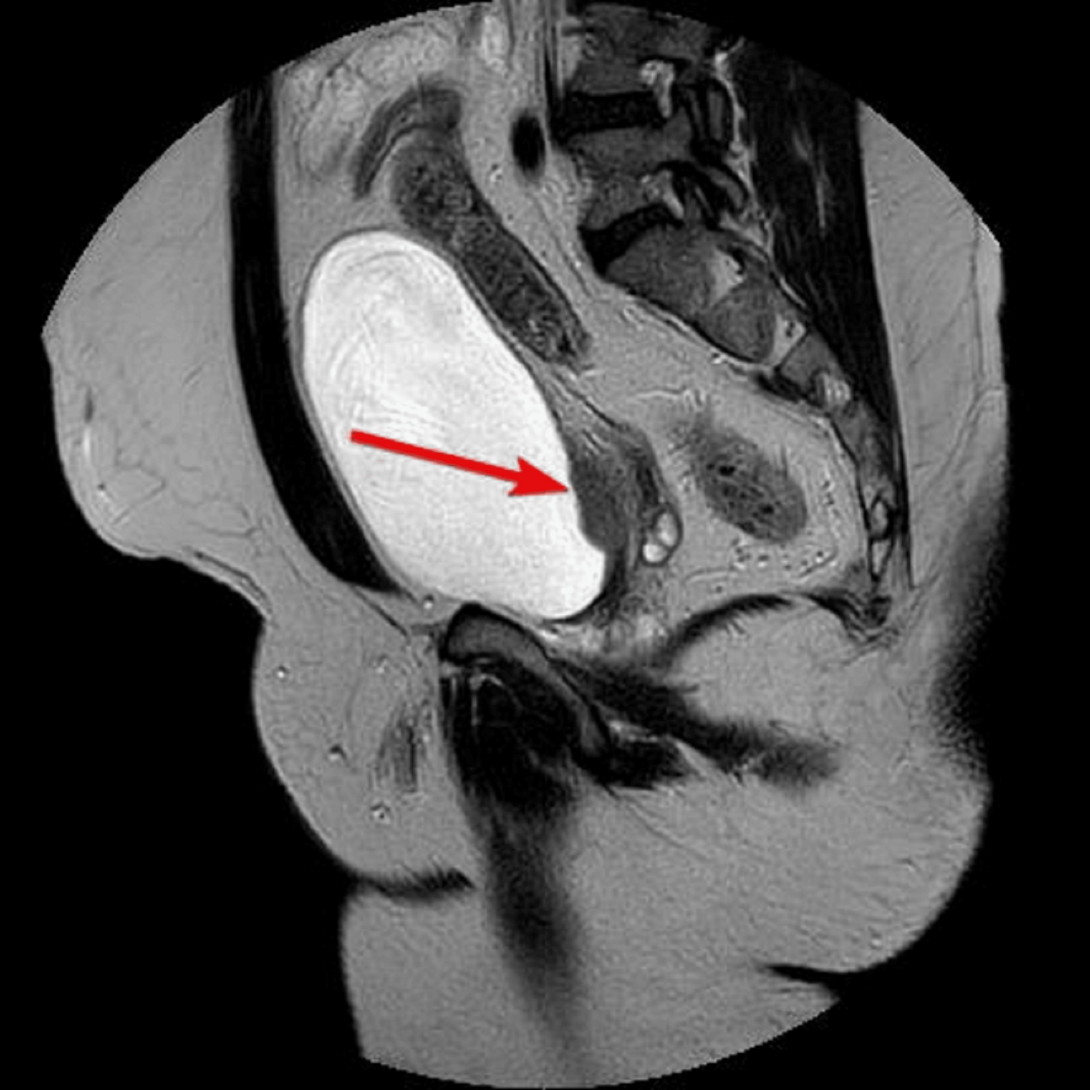
Lesion along the right bladder base visualized on MRI T2 sagittal images.

**Figure 3:**
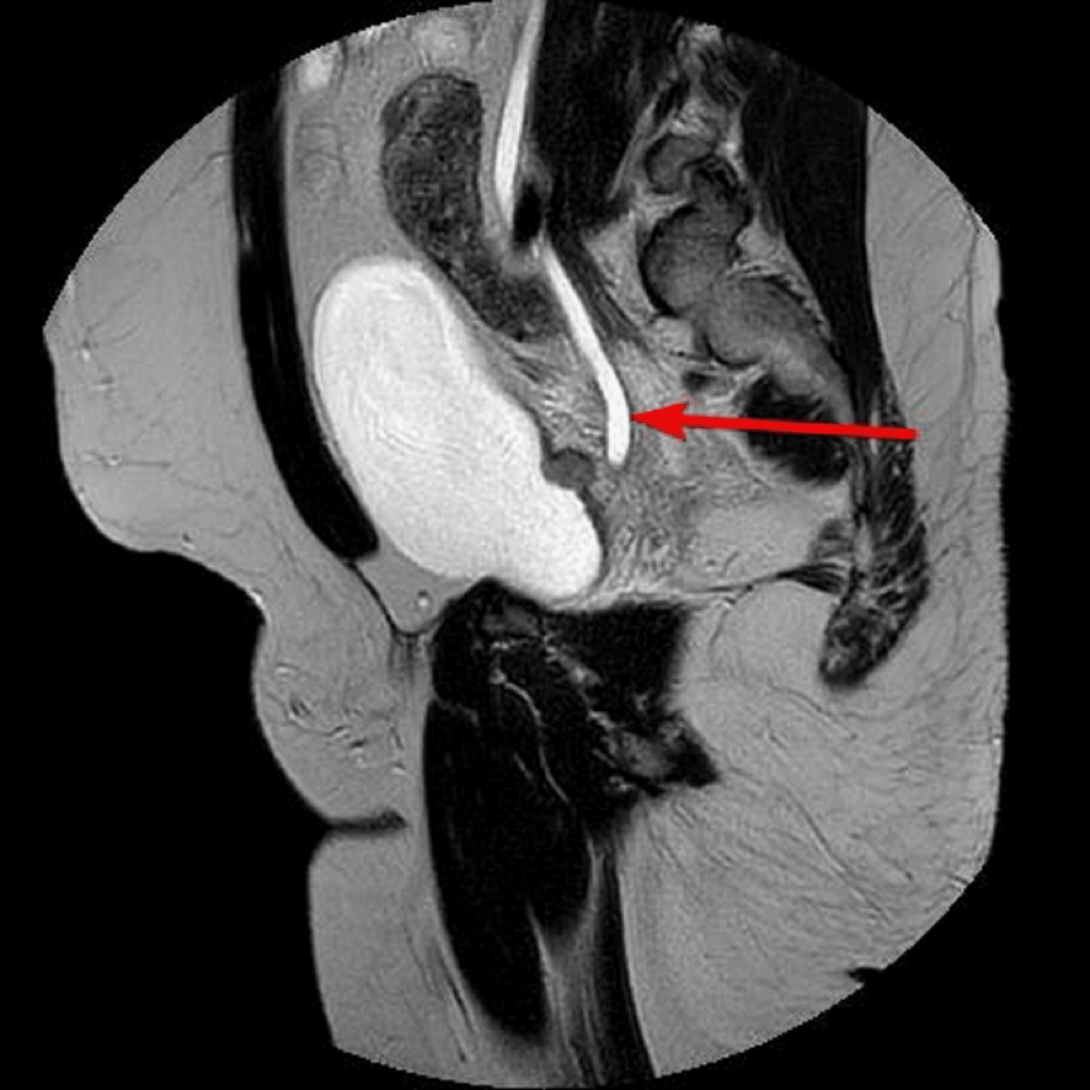
Right hydroureter appreciated on MRI.

A 46-year-old male with no comorbidities, who was a nonsmoker, had no family history of carcinoma, and had worked in the police department, presented at the urology clinic with a two-month history of painless hematuria, urinary urgency, dysuria, and nocturia. The patient had no history of weight loss, loss of appetite, or any abdominal pain. The patient was managed outside the hospital for a urinary tract infection for the last two months. The patient’s complete blood count was unremarkable, but deranged renal function tests with a serum creatinine level of 1.55 mg/dL and urine for routine examination revealed hematuria. Ultrasound of the abdomen and pelvis revealed right-sided hydronephrosis. Further imaging and MRI of the pelvis revealed nodular lesions along the right bladder base/ureterovesical junction causing a right hydronephroureter (Figures 1-3).

**Figure 4:**
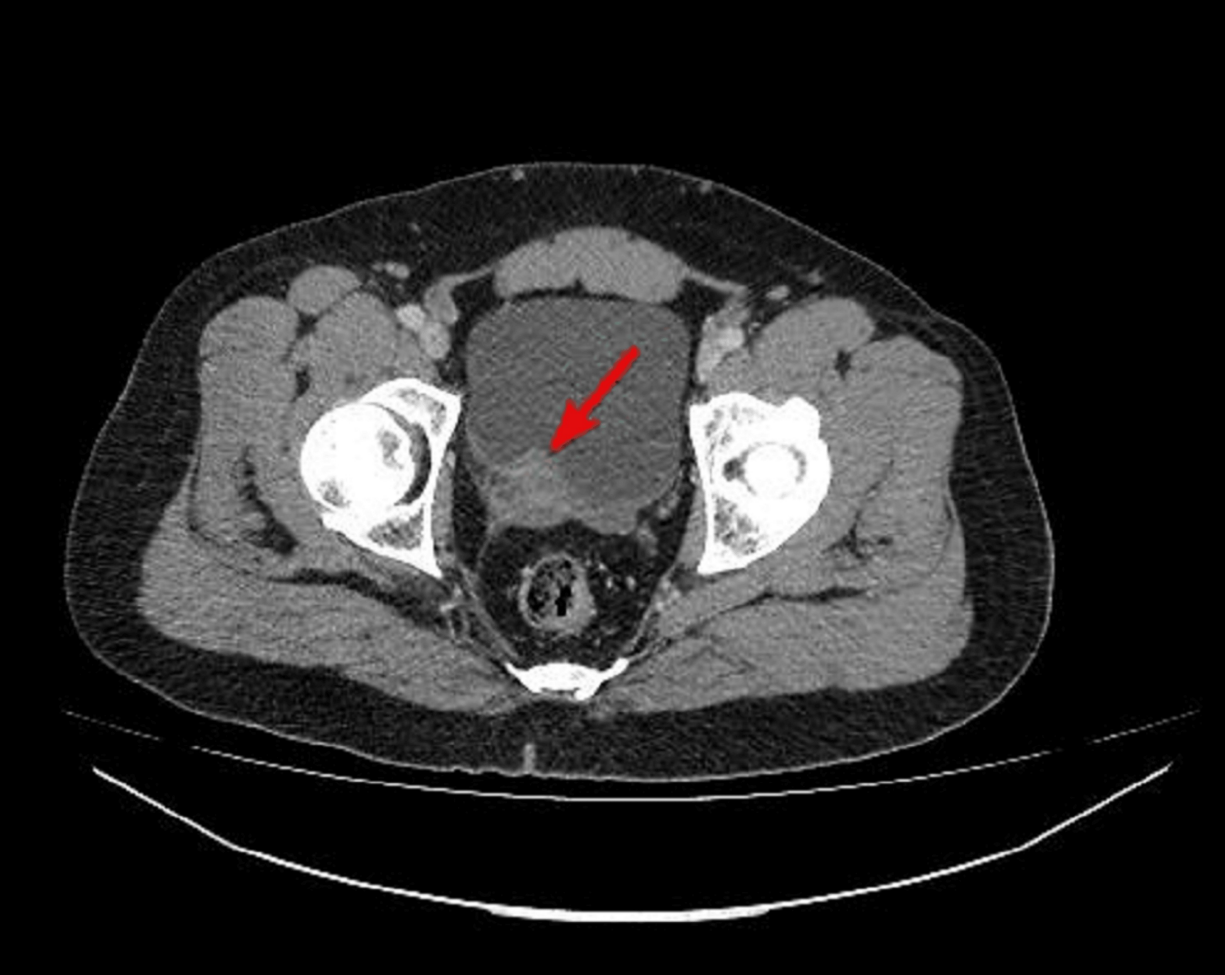
Soft tissue density mass involving the right posterior wall of the urinary bladder.

CT staging revealed a soft tissue density mass involving the right posterior wall of the urinary bladder causing the right hydronephroureter, stage T3bN1M0 (Figure 4).

**Figure 5:**
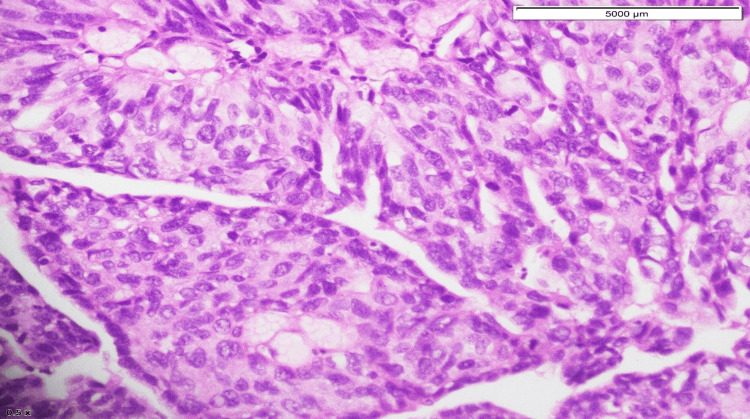
Scattered sheets of cells with high-grade plasmacytoid features.

**Figure 6:**
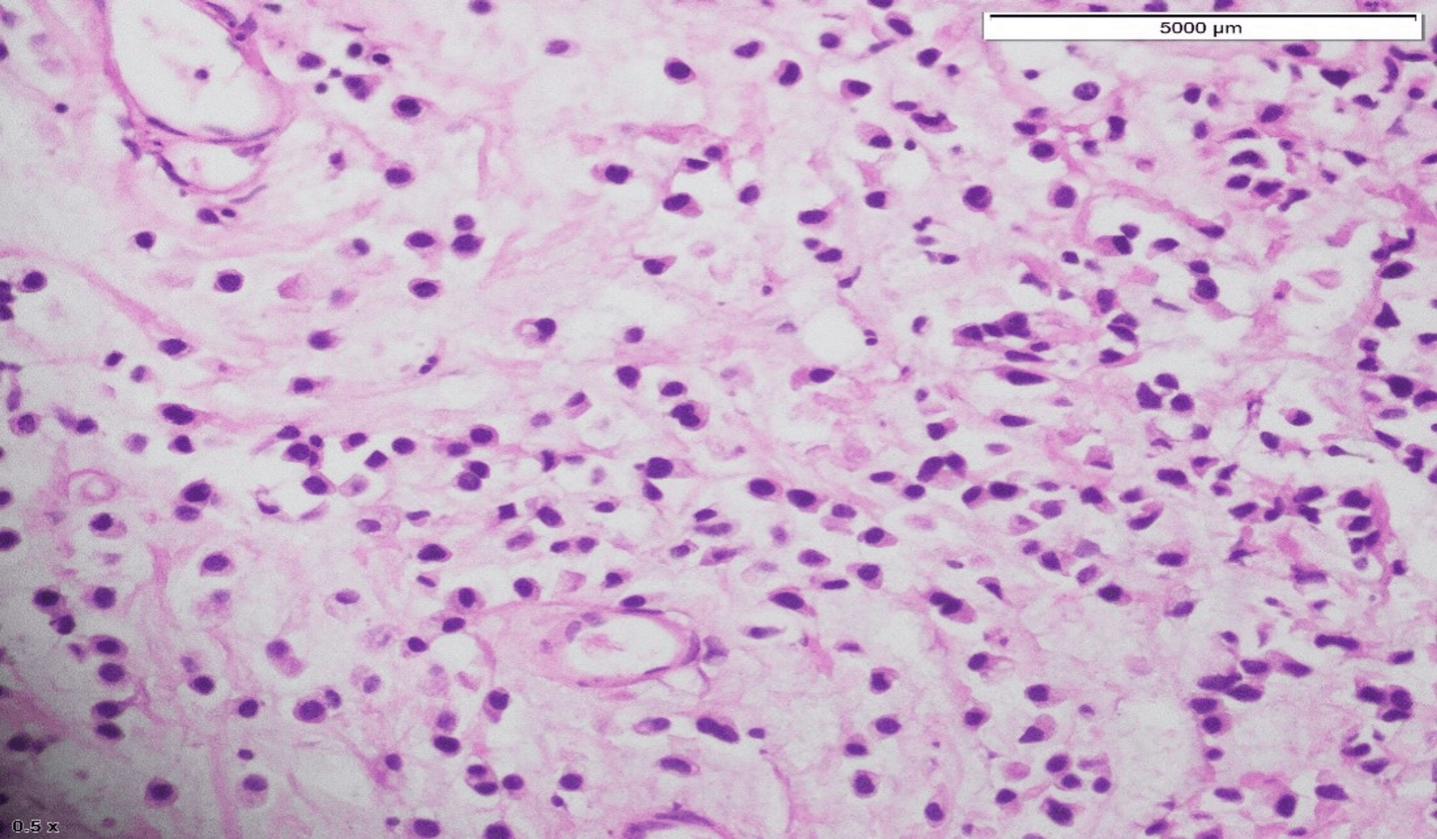
Cells with abundant cytoplasm, with the nucleus eccentrically placed.

**Figure 7:**
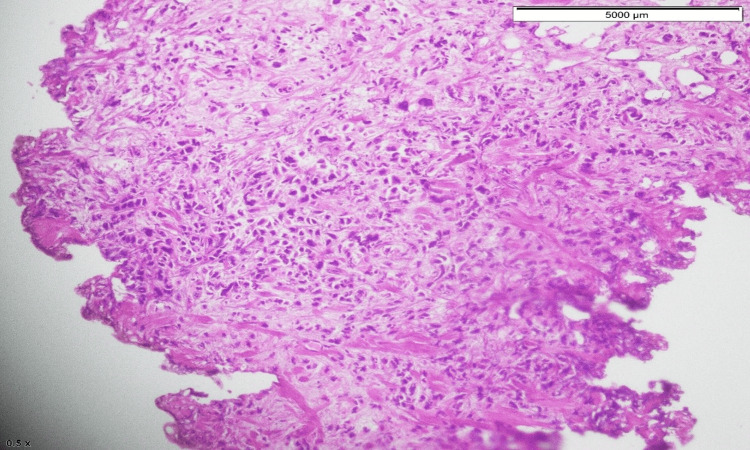
Diffuse nested and discohesive pattern of tumor cells.

The patient then underwent cystoscopy and transurethral resection of the bladder tumor. Histopathology of the specimen revealed high-grade papillary urothelial carcinoma (with plasmacytoid differentiation). The tumor involved the lamina propria and detrusor muscle (pT2) (Figures 5-7).

**Figure 8:**
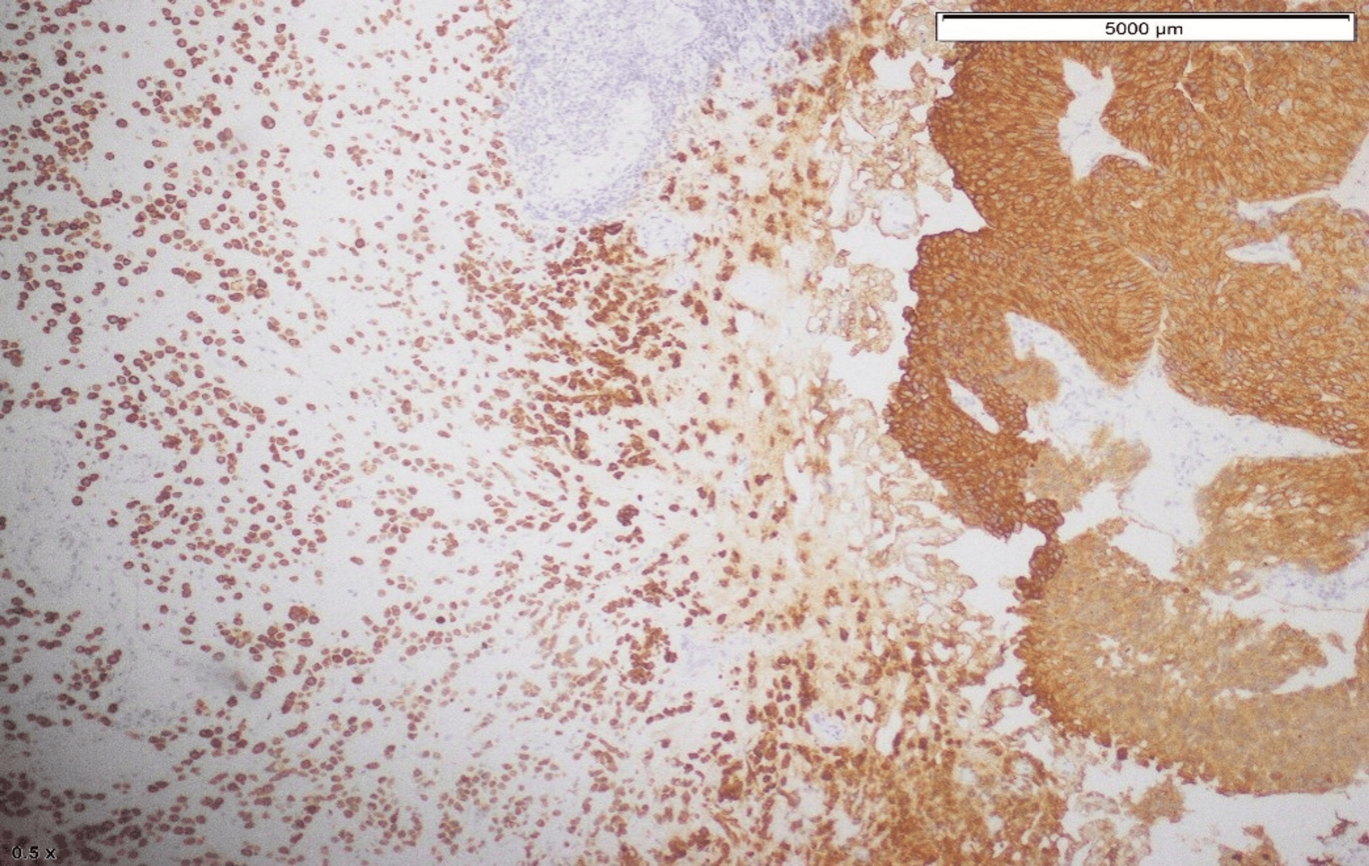
GATA3 positivity in tumor cells.

Tumor cells were positive for GATA3 and CD138 (Figure 8).

**Figure 9:**
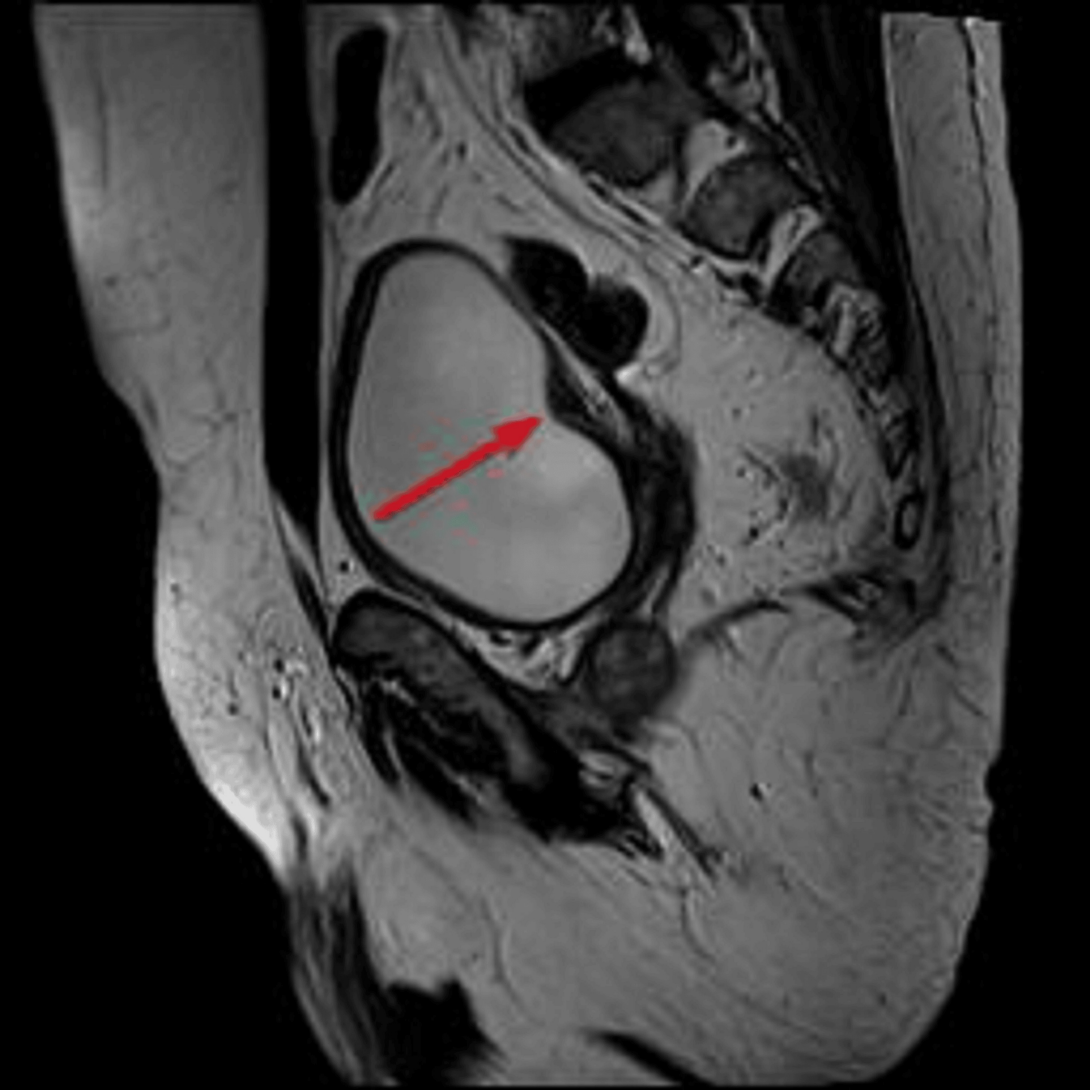
Resolved bladder wall lesion can be seen in MRI T2 sagittal images.

**Figure 10:**
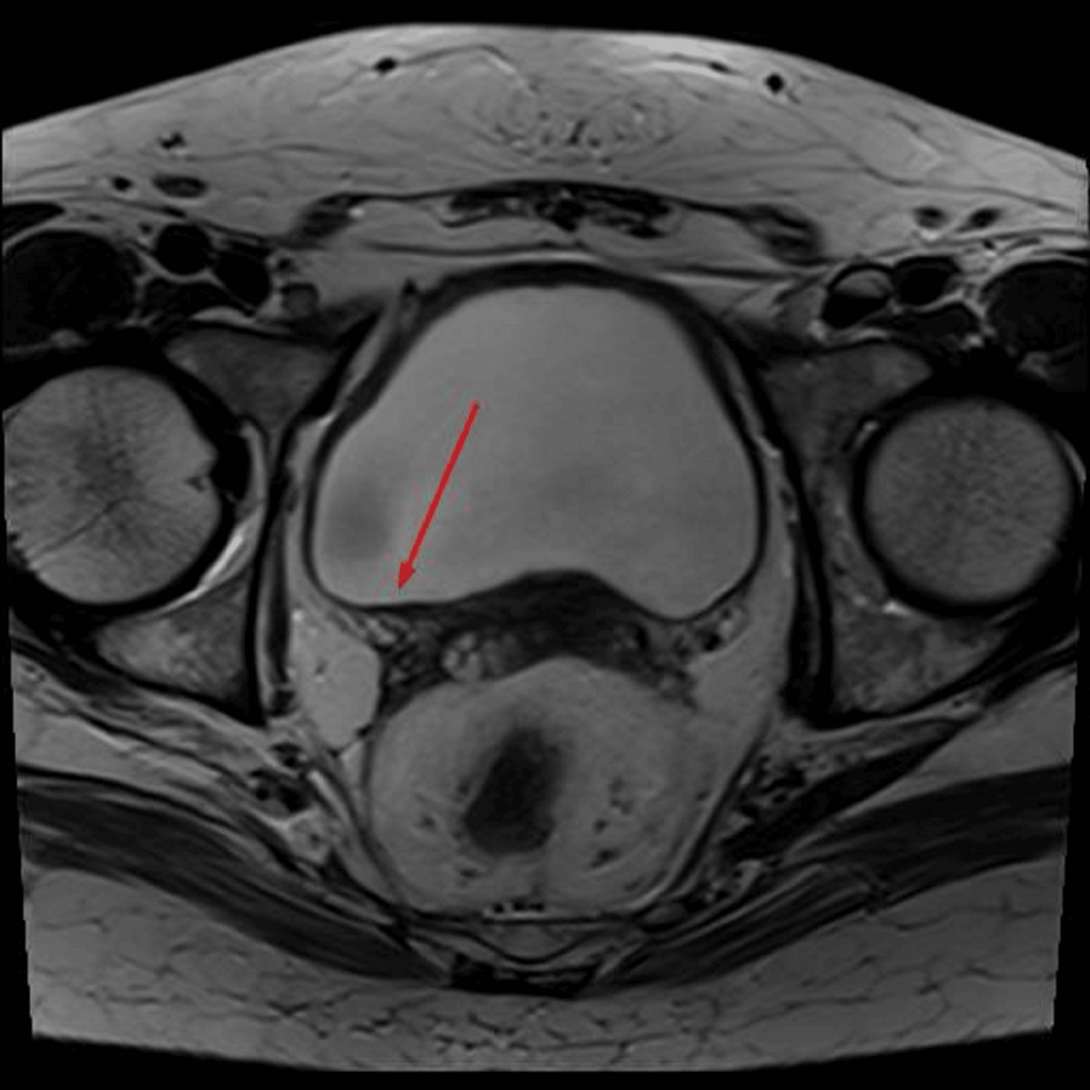
Resolved bladder wall lesion can be seen in MRI T2 images.

The patient’s case was discussed at our multidisciplinary team meeting, and it was decided to perform a right-sided nephrostomy tube insertion, followed by neoadjuvant chemotherapy and surgical assessment. The patient received dose-dense MVAC (methotrexate, vinblastine, doxorubicin, cisplatin) chemotherapy with a 50% dose reduction for cisplatin in the first two cycles due to low estimated glomerular filtration rate and creatinine clearance. A full dose of chemotherapy was given in the third and fourth cycles of chemotherapy as the creatinine clearance rate returned to normal. The patient was admitted to the intensive care unit 72 hours after cycle four of chemotherapy due to grade IV cytopenias, neutropenic colitis, and electrolyte imbalances. The patient’s functional status decreased, and his Eastern Cooperative Oncology Group score decreased from 0 to 1. Cycle five of chemotherapy was given with 25% dose reductions of all drugs. The patient subsequently required inpatient admission for febrile neutropenia and grade II cytopenias. Further chemotherapy was stopped due to the side effect profile and drop in performance status. The patient underwent repeat imaging for response assessment. A pelvic MRI revealed interval resolution of previously observed urinary bladder masses along the right basal and posterolateral walls (Figures 9, 10).

The right distal ureter was locally dilated; however, the proximal ureter was not dilated. CT of the chest, abdomen, and pelvis revealed no distant nodal or visceral metastasis. The patient was rediscussed in a multidisciplinary team meeting and underwent cystectomy with an ileal conduit as per recommendations. The patient is currently undergoing urological follow-up.

## Discussion

Plasmacytoid bladder tumors account for 1-4.9% of all invasive urothelial carcinomas. This tumor is more common in elderly males with a mean age at diagnosis between 62 and 66 years. Risk factors for this variant include smoking history and male sex. Patients with this diagnosis usually present with advanced-stage disease compared to those with conventional urothelial carcinomas. The 2016 WHO classification of urothelial tract tumors included plasmacytoid variants of infiltrative urothelial tumors. It may show diffuse to extensive infiltration of the bladder, which presents as a “linitis plastica,” as it thickens the bladder wall. The tumor had extensive involvement of the bladder wall and was then visualized via cystoscopy during examination and sampling. Histopathologically, tumor cells have characteristic cytological features. The tumor cells are round to polygonal with abundant cytoplasm and the nucleus is usually present eccentrically [7]. Tumor cells are scattered in the form of clusters, along with nests of non-cohesive cells that infiltrate into the bladder wall. Histopathology slides of this patient had a similar cellular pattern. Focal distribution of mucin-containing cells can be present in PUCs which may mimic signet ring cells. This kind of plasmacytoid morphology is observed in various lymphomas and plasmacytomas and can lead to diagnostic challenges [8].

Thus, immunohistochemical staining helps further narrow the diagnosis. CK and CK7 help identify the epithelial origin of the transitional cell type. Furthermore, GATA3, p63, S100P, CK20, and uroplakin II are markers of urothelial carcinoma. Another marker that is common in both plasmacytoma and urothelial carcinoma is CD138 [9]. However, PUC does not express kappa or lambda light chains, although plasmacytoma does, which helps differentiate between the two. However, we did not check kappa or lambda light chain expression in our patient. Fritsche et al. proposed that plasmacytoid differentiation in urothelial cancer may be linked to E-cadherin loss [10]. E-cadherin maintains cell adhesion, and loss of its expression leads to increased cellular invasiveness.

More than 50% of urothelial carcinomas invade muscularis propria and 30% have metastasized to lymph nodes at the time of presentation. Moreover, peritoneal involvement at presentation is also common (observed in 33% of tumors). When radical cystectomy is performed, invasion beyond muscularis propria is commonly detected.

As it is a rare tumor, treatment is not well defined, but the treatment approach is similar to that for other urothelial carcinomas, including surgery, chemotherapy, and radiotherapy [11]. These tumors are aggressive and have a poor prognosis. The median overall survival of PUC was reported to be 3.8 years in one of the case series from Memorial Sloan Kettering Cancer Center whereas the overall survival of the usual urothelial carcinomas was eight years. There is also poorer cancer-specific survival and local recurrence survival with the plasmacytoid variant of urothelial carcinomas compared to other urothelial carcinomas, which was reported in a series from the Mayo Clinic.

## Conclusions

The plasmacytoid variant of urothelial carcinoma is a rare variant that is an aggressive subset of tumors with a poor prognosis. A thorough histopathological evaluation of the specimen helps to lead to a definitive diagnosis with the help of immunohistochemical staining, including a broad panel of antibodies. Identification of this rare entity is necessary to initiate early, appropriate, and aggressive treatment. Treatment is not well defined, but downstaging is achieved with platinum-based neoadjuvant chemotherapy. Only a few survivors have been reported to date.

## References

[1] Burger M, Catto JW, Dalbagni G (2013). Epidemiology and risk factors of urothelial bladder cancer. Eur Urol.

[2] Ferlay J, Colombet M, Soerjomataram I (2018). Cancer incidence and mortality patterns in Europe: estimates for 40 countries and 25 major cancers in 2018. Eur J Cancer.

[3] Berdik C (2017). Unlocking bladder cancer. Nature.

[4] Aben KK, Witjes JA, Schoenberg MP, Hulsbergen-van de Kaa C, Verbeek AL, Kiemeney LA (2002). Familial aggregation of urothelial cell carcinoma. Int J Cancer.

[5] Sahin AA, Myhre M, Ro JY, Sneige N, Dekmezian RH, Ayala AG (1991). Plasmacytoid transitional cell carcinoma. Report of a case with initial presentation mimicking multiple myeloma. Acta Cytol.

[6] Compérat EM, Burger M, Gontero P (2019). Grading of urothelial carcinoma and the new "World Health Organisation Classification of Tumours of the Urinary System and Male Genital Organs 2016". Eur Urol Focus.

[7] Wang Z, Lu T, Du L (2012). Plasmacytoid urothelial carcinoma of the urinary bladder: a clinical pathological study and literature review. Int J Clin Exp Pathol.

[8] Rahman K, Menon S, Patil A, Bakshi G, Desai S (2011). A rare case of plasmacytoid urothelial carcinoma of bladder: diagnostic dilemmas and clinical implications. Indian J Urol.

[9] Nigwekar P, Tamboli P, Amin MB, Osunkoya AO, Ben-Dor D, Amin MB (2009). Plasmacytoid urothelial carcinoma: detailed analysis of morphology with clinicopathologic correlation in 17 cases. Am J Surg Pathol.

[10] Fritsche HM, Burger M, Denzinger S, Legal W, Goebell PJ, Hartmann A (2008). Plasmacytoid urothelial carcinoma of the bladder: histological and clinical features of 5 cases. J Urol.

[11] Sorce G, Flammia RS, Hoeh B (2022). Plasmacytoid variant urothelial carcinoma of the bladder: effect of radical cystectomy and chemotherapy in non-metastatic and metastatic patients. World J Urol.

